# The impact of athlete burnout on academic burnout among college athletes: a multiple mediation model based on emotion regulation and sleep

**DOI:** 10.3389/fpsyg.2025.1669344

**Published:** 2025-09-16

**Authors:** Guihua Xu, Xirui Yang, Qiyong Zhang, Chenyang Li

**Affiliations:** ^1^College of Physical Education, Yangzhou University, Yangzhou, China; ^2^The College of Educational Science, Yangzhou University, Yangzhou, China

**Keywords:** college athletes, athlete burnout, academic burnout, emotion regulation, sleep quality

## Abstract

**Background:**

College athletes often face dual demands from training and academics, increasing their risk of both athletic and academic burnout. However, the transfer mechanism between these two forms of burnout remains underexplored, especially regarding the roles of emotional regulation and sleep.

**Methods:**

A cross-sectional survey was conducted among 1,918 college athletes using validated self-report measures. Structural equation modeling was applied to examine the direct and indirect effects of college athlete burnout on academic burnout, with cognitive reappraisal and sleep quality as mediators.

**Results:**

College athlete burnout significantly predicted academic burnout (β = 0.412, *p* < 0.001). Both cognitive reappraisal (β = 0.071, *p* < 0.001) and sleep quality (β = 0.037, *p* < 0.001) partially mediated this relationship. Additionally, a significant chain mediation pathway was identified through cognitive reappraisal and sleep quality (β = 0.006, *p* < 0.01), accounting for 1.14% of the total effect.

**Conclusion:**

Athlete burnout can spill over into academic burnout among college athletes. Beyond confirming this relationship, the study highlights the critical roles of emotional regulation and sleep quality as mechanisms linking the two domains. These findings provide theoretical insight into burnout transfer and underscore the importance of integrating psychological skills training and sleep management into interventions aimed at supporting student-athletes' academic adjustment and mental health.

## 1 Introduction

In the context of increasingly competitive and complex higher education, college students are experiencing unprecedented academic pressure and psychological challenges (Piredda et al., 2025). Heavy academic load, unclear learning goals, and declining motivation are gradually contributing to a widely recognized psychological condition among students—academic burnout (Fatima et al., 2024). Theoretically, academic burnout is a negative psychological state resulting from prolonged depletion of psychological resources, reflecting a maladaptive response to chronic academic stress (Schaufeli et al., 2002). According to Schaufeli et al.'s three-dimensional model of academic burnout, the concept comprises three core dimensions: emotional exhaustion, cynicism, and reduced academic efficacy (Schaufeli et al., 2002). Unlike transient fatigue, academic burnout is a persistent depletion process that significantly impairs students' motivation, cognitive functioning, and mental wellbeing (May et al., 2015). Studies have shown that academic burnout not only undermines academic performance but also harms psychological and physical health, increasing risks of anxiety, depression, and even suicidal ideation (Sierpina, 2005; Andargeery et al., 2025). Therefore, exploring the causes and mechanisms of academic burnout has become a key issue in educational psychology, particularly among special student groups like collegiate athletes who bear multiple psychological burdens.

Against the backdrop of widespread academic burnout, collegiate athletes—who juggle both academic and athletic roles—face more complex psychological burdens, warranting greater attention. In contrast to their non-athlete peers, collegiate athletes are required to simultaneously fulfill academic obligations and endure the rigorous demands of athletic training and competitions, making them particularly vulnerable to the chronic consumption of psychological resources under this dual-pressure context (Gustafsson et al., 2008). Consequently, among student-athletes, athlete burnout represents another prominent concern alongside academic burnout that warrants careful attention. According to Raedeke's (1997) cognitive-affective stress model, athlete burnout is a chronic psychological syndrome caused by prolonged training and competition stress, and includes three dimensions: emotional/physical exhaustion, sport devaluation, and reduced sense of accomplishment (Raedeke, 1997). This model emphasizes that when athletes consistently perceive their efforts as disproportionate to the rewards received, and are unable to regulate stress effectively, they are prone to negative cognitive appraisals and emotional reactions, ultimately resulting in burnout (Raedeke, 1997). Prior research has demonstrated that different forms of burnout tend to exhibit similar patterns across diverse contexts (Schaufeli and Enzmann, 2020). Importantly, both athlete burnout and academic burnout have been shown to not only undermine mental health and academic achievement but also negatively affect athletic performance and emotion regulation capacity (Sadeh et al., 2002; Gerber et al., 2013). Nevertheless, systematic investigations remain scarce regarding whether athlete burnout can transfer to the academic domain, thereby heightening the risk of academic burnout among college athletes.

Although previous studies have found that athlete burnout and academic burnout are each associated with various negative psychological outcomes (Gustafsson et al., 2017; Zhang et al., 2007), the relationship between the two and their underlying mechanisms remain unclear. To gain a deeper understanding of the potential transfer of burnout experienced by college athletes across the dual contexts of training and academic learning, it is necessary to introduce key psychological and physiological resource variables for further exploration at the mechanistic level. Existing research has provided preliminary evidence for the phenomenon of burnout transfer across different life domains. For example, Schaufeli et al. found that occupational burnout caused by work-related stress can extend into the home domain, leading to what is referred to as “home burnout” (Peeters et al., 2005). Similarly, Salmela-Aro et al. reported that emotional exhaustion and reduced efficacy experienced in the academic context may transfer to cultural, leisure, and social domains, thereby diminishing individuals' overall wellbeing and their ability to sustain interest and engagement (Salmela-Aro et al., 2022). According to the Conservation of Resources (COR) theory, individuals who experience resource depletion in one domain may suffer functional impairment in other domains if timely replenishment does not occur (Hobfoll, 1989). For college athletes in particular, limited psychological resources must be allocated between both training and academic contexts. When emotional or physical exhaustion accumulates in the athletic setting without adequate recovery, it may “transfer” into the academic domain, manifesting as cognitive fatigue, reduced motivation, or diminished academic efficacy—namely, academic burnout (Sorkkila et al., 2018). Within this framework, emotion regulation ability and sleep quality are considered key mechanisms for maintaining or restoring psychological resources. These factors may serve as mediators in the relationship between Athlete Burnout and academic burnout among college athletes.

First, emotion regulation refers to the process by which individuals manage and adjust their emotional states. It primarily involves two strategies: Cognitive Reappraisal (CR) and Expressive Suppression (ES), and is considered a core psychological resource for maintaining mental health and situational adaptability (Gross, 2002). According to Gross's emotion regulation theory, emotion regulation is a process through which individuals use cognitive and behavioral strategies to modulate emotional responses (Gross, 1998). Among these, CR is widely recognized as one of the most effective and adaptive strategies (Gross, 2002). CR involves altering one's interpretation of an emotional event in order to change its emotional impact (Gross, 1998). By reframing the meaning of the event at an early stage of the emotional response, this strategy effectively reduces the intensity of negative emotions while enhancing the experience of positive emotions (Xu B. et al., 2023). Research has shown that individuals with lower emotion regulation ability are more prone to experiencing negative emotional responses such as anxiety and depression when facing stress and fatigue. These emotional reactions, in turn, can undermine academic motivation and exacerbate academic burnout (DeLongis et al., 1988). In contrast, individuals with stronger emotion regulation skills are more capable of using positive CR strategies to reframe their understanding of challenging situations, thereby mitigating the negative psychological effects of burnout and maintaining academic engagement—even under high-pressure conditions (Gross, 2015). For college athletes, the ongoing accumulation of Athlete Burnout not only impairs performance in competitive settings but may also diminish their fundamental capacity for emotion regulation. Studies have found that athletes exposed to prolonged high-intensity training without adequate recovery support tend to exhibit weakened emotion regulation strategies—such as less frequent use of CR and increased reliance on less effective strategies like ES—which can further intensify emotional exhaustion and psychological distress (Xin et al., 2024; Van Slingerland, 2016). For example, a study by Lu et al. found that higher levels of Athlete Burnout were associated with a lower frequency of using positive emotion regulation strategies and significantly reduced emotional recovery capacity (Lin et al., 2022). Therefore, under the influence of Athlete Burnout, emotion regulation ability may serve as a key mediating mechanism in the relationship between Athlete Burnout and academic burnout among college athletes.

Secondly, Sleep Quality, as a core physiological recovery resource, plays an irreplaceable role in alleviating cognitive load, regulating emotions, and restoring bodily functions (Vandekerckhove and Wang, 2017). According to recovery theory, individuals must rely on high-quality sleep to replenish their resources after experiencing stress during the day in order to maintain optimal functioning (Sonnentag and Fritz, 2007). Chronic sleep deprivation or poor sleep quality can lead to the depletion of both physiological and psychological resources, resulting in increased fatigue, emotional instability, and impaired cognitive functioning—all of which elevate the risk of academic burnout (Pilcher and Huffcutt, 1996; Abbasmanesh et al., 2019). Empirical studies have shown that sleep quality is closely associated with attention, executive functioning, learning capacity, and memory consolidation—cognitive abilities that are essential for academic performance in college students (Hirshkowitz et al., 2015). For college athletes, Athlete Burnout has also been shown to negatively affect sleep quality (Renaldo, 2022). Existing research indicates that emotional exhaustion and cognitive overload resulting from high-intensity training can significantly disrupt sleep structure and the sleep initiation process, manifesting in issues such as prolonged sleep onset latency and increased nighttime awakenings (Brand et al., 2010). In particular, when athletes are unable to mentally detach from the pressures of training or competition, they may experience a sustained state of hyperarousal, which can further contribute to the development of sleep disturbances (Leeder et al., 2012). Therefore, sleep quality not only reflects an individual's ability to recover from external stressors but may also serve as a critical pathway through which Athlete Burnout transfers across domains and manifests as academic burnout.

Finally, existing research suggests that individual differences in emotion regulation ability may influence sleep quality, indicating a potential intrinsic link between emotional regulation and sleep (Vanek et al., 2020). According to Gross's emotion regulation theory, strategies such as CR can be employed before the onset of negative emotional responses, thereby reducing the sustained activation of negative emotions (Gross, 1998). Such prolonged emotional activation has been identified as a key mechanism underlying pre-sleep psychological arousal, delayed sleep onset, and sleep fragmentation (Palmer and Alfano, 2017). Mauss et al. found that individuals with poor emotion regulation abilities are more likely to experience nighttime emotional rumination, anxiety, and physiological arousal, all of which significantly impair sleep quality (Mauss et al., 2013). This chain pathway has received empirical support. For example, in a study of adolescents, Brand et al. (2016) found that sleep quality served as a significant mediator in the relationship between emotion regulation ability and levels of anxiety and depression, forming a chain model of emotion regulation → sleep quality → anxiety/depression (Brand et al., 2016). Accordingly, under the dual pressures of training and academics faced by college athletes, effective emotion regulation strategies may not only directly buffer the emotional exhaustion resulting from Athlete Burnout, but may also indirectly reduce the risk of Academic Burnout by promoting sleep—a key physiological recovery mechanism.

**Figure 1:**
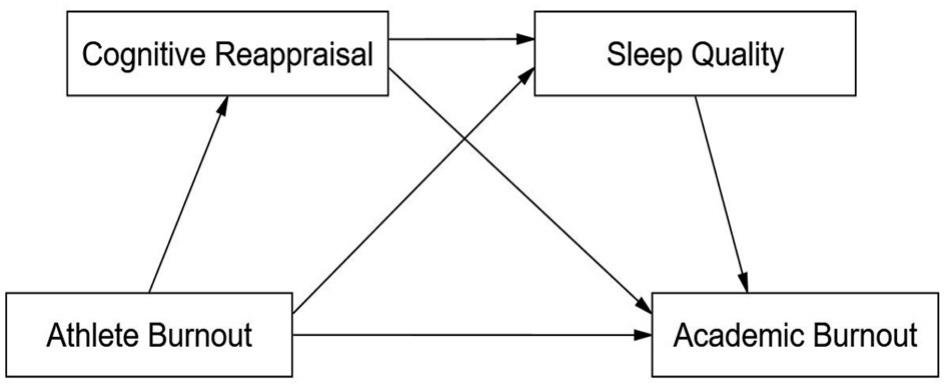
Hypothetical multiple mediation model of CR and sleep quality in the relationship between athlete burnout and academic burnout of college athletes.

In summary, although previous studies have examined athlete burnout and academic burnout separately, the interrelationship between the two and the mechanisms underlying their cross-domain transfer remain insufficiently explored among college athletes. Investigating this relationship and its mechanisms may not only contribute to elucidating the psychological processes behind burnout transfer but also provide theoretical guidance and practical implications for enhancing academic adjustment and mental health in this population. Based on existing theories and empirical research, this study proposes a multiple mediation model (see Figure 1) to systematically examine the relationship between Athlete Burnout and Academic Burnout among college athletes, with a specific focus on the mediating roles of emotion regulation and sleep quality. Accordingly, the following research hypotheses are proposed:

Hypothesis 1 (H1): Athlete Burnout significantly and positively predicts Academic Burnout among college athletes.
Hypothesis 2 (H2): Emotion regulation ability partially mediates the relationship between Athlete Burnout and Academic Burnout.
Hypothesis 3 (H3): Sleep quality partially mediates the relationship between Athlete Burnout and Academic Burnout.
Hypothesis 4 (H4): Emotion regulation ability and sleep quality jointly form a sequential (chain) mediation pathway between Athlete Burnout and Academic Burnout.

This study aims to explore the underlying mechanisms through which Athlete Burnout influences Academic Burnout, with a specific focus on the mediating roles of emotion regulation ability and sleep quality. By constructing a multiple mediation model, the study seeks to deepen the understanding of the relationship between Athlete Burnout and Academic Burnout among college athletes. Furthermore, it provides a scientific basis for academic management and mental health interventions targeting this population. The findings will help inform more balanced strategies between training and academic responsibilities, promote more effective recovery patterns, and enhance academic achievement. In addition, the study offers empirical support for the improvement of physical education programs and mental health support systems for student-athletes, thereby contributing to the sustainable development of collegiate sports.

## 2 Methods

### 2.1 Participants and research design

This study was conducted from November to December 2024 using a cross-sectional research design. Participants were recruited through convenience sampling from university sports teams across more than 20 universities in China. To ensure high response quality and participant engagement, we collaborated with faculty coordinators at each institution to distribute paper-based questionnaires directly to student-athletes. Investigators provided on-site guidance and addressed any questions participants had during the survey process. Prior to participation, all individuals were informed of the study's purpose and procedures, as well as their right to withdraw at any time. A total of 2,115 questionnaires were distributed, of which 1,967 were returned (response rate: 93%). After data screening, 1,918 valid questionnaires were retained (validity rate: 97.5%). Among the valid respondents, 1,262 were male and 656 were female, with a mean age of 19.97 years (SD = 1.4). In terms of residence, 500 participants (26.1%) were from rural areas, 218 (11.4%) from towns, 288 (15.0%) from county-level cities, and 912 (47.5%) from urban areas. Regarding family structure, 1,660 participants (86.5%) came from intact families, 88 (4.6%) from blended families, 161 (8.4%) from single-parent families, and 9 (0.5%) were orphans. In terms of sports categories, there were 1,089 athletes in combat sports (boxing, taekwondo, karate etc.), 494 athletes in ball games (football, basketball, volleyball etc.), 213 athletes in athletics (javelin, 800 m, long jump etc.), 56 athletes in water sports (swimming, canoeing etc.), and 66 athletes in other sports (archery, cycling etc.). In terms of recent athletic performance, 391 athletes won championships in provincial or higher-level competitions, 276 athletes achieved second place, 242 athletes secured third place, 332 athletes ranked between fourth and fifteenth place, and 677 athletes either did not participate in recent competitions or did not obtain a ranking.

### 2.2 Instruments

#### 2.2.1 Athlete burnout questionnaire

Athlete burnout was assessed using the Athlete Burnout Questionnaire (ABQ) developed by Raedeke et al. and revised by Zhang Liwei et al. for use in Chinese populations (Zhang and Ma, 2010; Raedeke and Smith, 2001). The ABQ consists of 15 items divided into three dimensions: emotional/physical exhaustion, reduced sense of accomplishment, and sport devaluation. Each item is rated on a 5-point Likert scale ranging from 1 (“never”) to 5 (“always”). The total score is calculated by summing the responses to all 15 items, with higher scores indicating a higher level of athlete burnout. In the current study, the ABQ demonstrated good internal consistency, with a Cronbach's alpha coefficient of 0.869.

#### 2.2.2 Learning burnout scale for university students

This study employed the Learning Burnout Scale for Undergraduates (LBUS) to assess feelings of academic burnout experienced by college athletes in the learning context (Rong et al., 2005; Chen et al., 2025). The LBUS consists of 20 items across three dimensions: emotional exhaustion, inappropriate behavior, and reduced sense of achievement. Responses are rated on a 5-point Likert scale ranging from 1 (“strongly disagree”) to 5 (“strongly agree”). The scale was developed based on both exploratory factor analysis and confirmatory factor analysis, ensuring its reliability and validity in the undergraduate population. Previous studies have demonstrated that the LBUS possesses good psychometric properties and is an effective tool for measuring academic burnout. In the present study, the Chinese version of the LBUS showed high internal consistency, with a Cronbach's alpha coefficient of 0.906.

#### 2.2.3 Pittsburgh sleep quality index

This study employed the Pittsburgh Sleep Quality Index (PSQI) to assess the sleep quality of collegiate athletes. The PSQI was originally developed by Buysse et al. (1989) and later translated and validated in Chinese by Xianchen and colleagues (Zarrabi et al., 2024; Liu et al., 1996). The scale consists of 18 items, covering seven components: subjective sleep quality, sleep latency, sleep duration, habitual sleep efficiency, sleep disturbances, use of sleeping medication, and daytime dysfunction. Each component is scored from 0 to 3, with the total PSQI score ranging from 0 to 21. Higher scores indicate poorer overall sleep quality. The PSQI has been widely used in studies examining sleep quality among athlete populations and has demonstrated satisfactory reliability and validit (Xu N. et al., 2023). In the current study, the internal consistency of the PSQI was acceptable, with a Cronbach's alpha coefficient of 0.709.

#### 2.2.4 Emotion regulation questionnaire

The Emotion Regulation Questionnaire (ERQ) was used to assess the emotional regulation abilities of college athletes. The scale includes two dimensions: CR and ES. The CR subscale consists of 6 items, and the ES subscale includes 4 items, for a total of 10 items. All items are rated on a 7-point Likert scale, with higher scores indicating a greater frequency of using the corresponding emotion regulation strategy (Gross and John, 2003). The Chinese version of the ERQ has been validated by Si Gangyan et al. among athlete and student populations in China and has been widely applied in research involving Chinese athletes (Chunqing et al., 2014; Suting et al., 2013). In the present study, the overall Cronbach's alpha coefficient of the scale was 0.828. The subscale coefficients were 0.894 for CR and 0.756 for ES, indicating good internal consistency.

### 2.3 Statistical analysis

#### 2.3.1 Data analysis

The data analysis in this study was conducted in two steps. First, descriptive statistics and Pearson correlation analyses were performed using SPSS 26.0 to examine the preliminary relationships among variables. Second, structural equation modeling (SEM) was conducted using AMOS 24.0 to assess the direct effect of athlete burnout on academic burnout, as well as the multiple mediating effects of CR and sleep quality. The mediation effects were tested using the bootstrap method with 5,000 resamples. A 95% confidence interval (CI) that does not include zero was considered evidence of a significant indirect effect. In all models, sociodemographic variables including gender, age, place of residence, and family type were included as control variables.

#### 2.3.2 Common method bias test

All data in this study were collected through self-report measures, which may raise concerns regarding common method variance (CMV). In line with the recommendations of Zhou and Long (2004), several procedural remedies were implemented during the survey process to reduce potential CMV (Zhou, 2004). These included ensuring participants' anonymity, informing them that the data would be used solely for academic research purposes, and incorporating reverse-coded items in the questionnaires. To further enhance the methodological rigor, Harman's single-factor test was conducted prior to formal data analysis. An unrotated principal component analysis was performed on all items of the measured variables. The results showed that 11 factors had eigenvalues greater than 1, accounting for a total of 57.23% of the variance. The first factor explained 19.761% of the variance, which is well below the critical threshold of 40%. Therefore, it can be concluded that there is no serious common method bias in this study.

## 3 Results

### 3.1 Descriptive statistics and correlation analysis

Table 1 presents the means, standard deviations, and Pearson correlation coefficients for all study variables. The results indicated that college athlete burnout was significantly positively correlated with both sleep quality and academic burnout (*r* = 0.24, *p* < 0.01; *r* = 0.43, *p* < 0.01, respectively), and significantly negatively correlated with CR (*r* = −0.29, *p* < 0.01). Additionally, CR was negatively correlated with both sleep quality and academic burnout (*r* = −0.18, *p* < 0.01; *r* = −0.32, *p* < 0.01, respectively), while sleep quality was positively correlated with academic burnout (*r* = 0.26, *p* < 0.01). These results suggest that college athlete burnout, CR, and sleep quality may exert practically significant effects on academic burnout among college athletes, providing preliminary evidence consistent with H1, which posits that athlete burnout positively predicts academic burnout.

**Table 1:** Descriptive statistics and Pearson correlations among variables (*N* = 1,918).

**Variables**	**M ±SD**	**1**	**2**	**3**	**4**	**5**	**6**	**7**	**8**
1. Age	19.97 ± 1.40	1							
2. Gender	1.34 ± 0.48	−0.10^**^	1						
3. Living	2.84 ± 1.27	−0.07^**^	0.06^**^	1					
4. Family	1.23 ± 0.61	0.01	0.01	−0.04	1				
5. ABQ	2.57 ± 0.57	0.08^**^	−0.07^**^	−0.07^**^	−0.01	1			
6. CR	4.88 ± 1.166	−0.01	−0.01	0.04	−0.02	−0.29^**^	1		
7. PSQI	0.73 ± 0.43	0.06^**^	0.08^**^	−0.04	0.05^*^	0.24^**^	−0.18^**^	1	
8. LUBS	2.66 ± 0.69	0.02	0.01	−0.04	0.04	0.43^**^	−0.32^**^	0.26^**^	1

^*^p < 0.05, ^**^p < 0.01.

### 3.2 Mediation effect test

**Figure 2:**
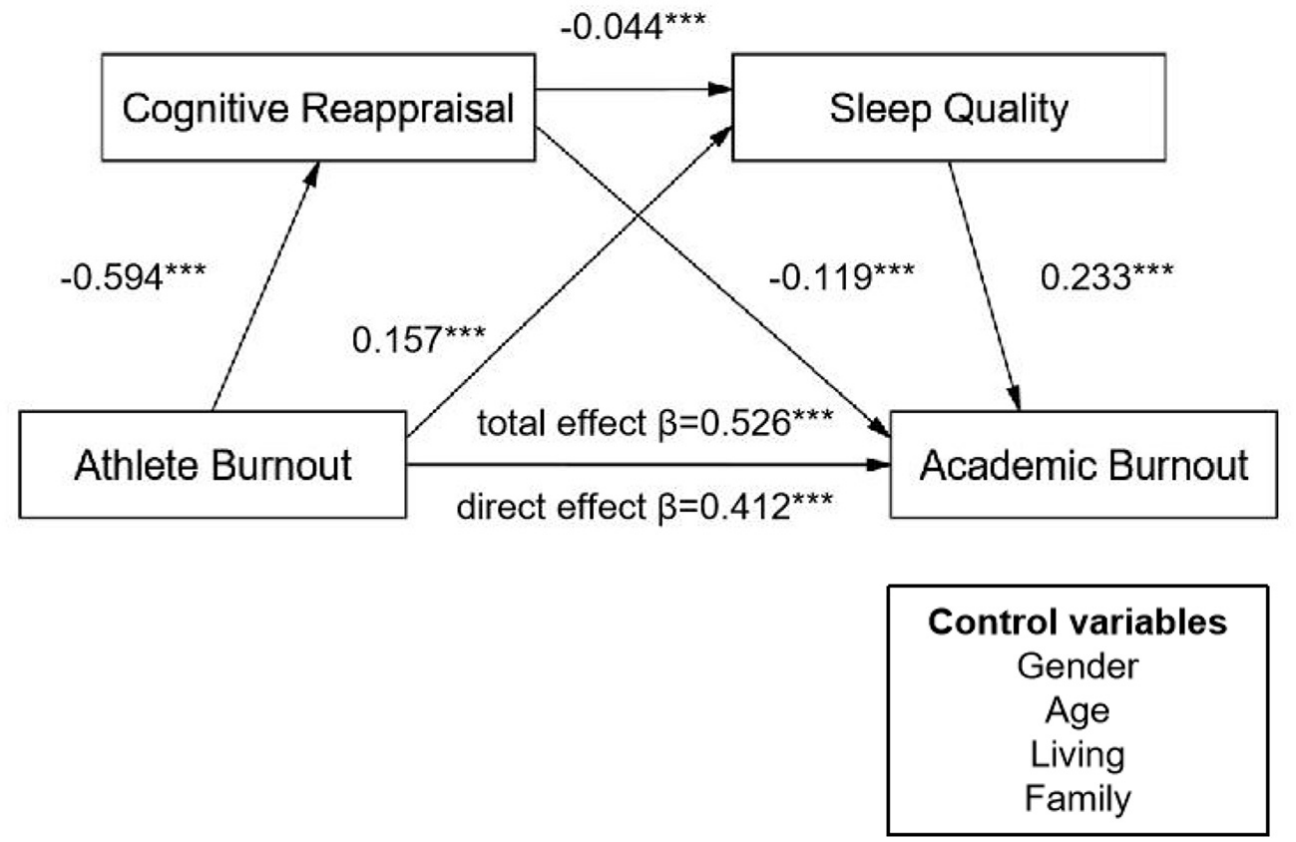
Mediating effect model of college athlete burnout on academic burnout. ***p < 0.001.

In this study, the structural equation model demonstrated good fit to the data: χ^2^/df = 4.191, GFI = 0.996, AGFI = 0.981, CFI = 0.996, TLI = 0.903, RMSEA = 0.041, and SRMR = 0.022. Gender, age, residence, and family background were included as control variables. College athlete burnout was set as the independent variable, academic burnout as the dependent variable, and CR and sleep quality as mediating variables to construct a structural equation model (Figure 2). The bias-corrected percentile Bootstrap method (with 5,000 resamples) was used to test the multiple mediation effects of college athlete burnout on academic burnout, and unstandardized effect value were reported.

As shown in Table 2, the direct effect of college athlete burnout on academic burnout was significant [β = 0.412, 95% CI [0.353, 0.471]], with the confidence interval not including zero, indicating that college athlete burnout significantly and positively predicts academic burnout. The direct effect accounted for 78.33% of the total effect, supporting H1. Single-path analysis showed that both CR and sleep quality played partial mediating roles in the relationship between college athlete burnout and academic burnout [β = 0.071, 95% CI [0.054, 0.089]; β = 0.037, 95% CI [0.024, 0.051]], with confidence intervals not containing zero. These indirect effects accounted for 13.50% and 7.03% of the total effect, respectively, providing support for H2 and H3, which proposed that CR and sleep quality partially mediate the relationship between athlete burnout and academic burnout.

**Table 2:** Bootstrap test results for the mediation effects of athlete burnout on academic burnout (*N* = 1,918).

**Path**	**β**	**Ratio**	**95% CI**			
			**Boot SE**	**Boot LLCL**	**Boot ULCL**	***P***
ABQ –> CR –> LUBS	0.071	13.50%	0.009	0.054	0.089	0
ABQ –> PSQI –> LUBS	0.037	7.03%	0.007	0.024	0.051	0
ABQ –> CR–> PSQI –> LUBS	0.006	1.14%	0.002	0.003	0.010	0
Total indirect effect	0.114	21.67%	0.011	0.091	0.137	0
Direct effect	0.412	78.33%	0.029	0.353	0.471	0
Total effect	0.526	100.00%	0.029	0.468	0.582	0

In addition, CR and sleep quality jointly formed a chain mediation pathway [β = 0.006, 95% CI [0.003, 0.010]], accounting for 1.14% of the total effect, thus supporting H4 that CR and sleep quality constitute a chain mediation pathway between college athlete burnout and academic burnout. These findings indicate that college athlete burnout not only directly affects academic burnout but also exerts indirect effects through both independent and sequential mediation mechanisms involving CR and sleep quality, highlighting the crucial roles of emotional regulation and sleep quality in the relationship between college athlete burnout and academic burnout.

## 4 Discussion

Grounded in the COR Theory, this study systematically explored the impact pathways through which college athlete burnout influences academic burnout, and for the first time, introduced CR and sleep quality as multiple mediators. The results revealed that college athlete burnout not only significantly and positively predicted academic burnout, but also exerted partial indirect effects through both CR and sleep quality. Moreover, a significant chain mediation effect was found between these two mediators. These findings clarify that the psychological resource depletion experienced in athletic training settings can transfer to academic domains, manifesting as academic burnout. They also highlight key regulatory mechanisms that help college athletes adapt to the dual pressures of training and studying. The present study enriches theoretical integration between sport psychology and educational psychology and provides actionable insights for the development of targeted interventions. Overall, the findings provide comprehensive support for H1–H4.

### 4.1 College athlete burnout and academic burnout

This study found that college athlete burnout significantly and positively predicts academic burnout, which is in line with Hypothesis 1, suggesting that the fatigue and psychological resource depletion accumulated in athletic training contexts may transfer to the academic domain. This transfer can trigger sustained exhaustion responses in terms of learning motivation, emotional state, and academic efficacy. The results align with previous research findings. For instance, Sorkkila et al., in a longitudinal study on Finnish high school athletes, observed a co-developmental trend between athletic and academic burnout, indicating that burnout experienced in one domain can migrate to another under dual academic and athletic demands (Sorkkila et al., 2018). Similarly, Ahola et al. reported that in medical students, prolonged academic pressure not only led to significant fatigue but also to cognitive decline. This exhaustion tended to persist across examination and study situations, implying a cross-situational loss of psychological resources (Ahola et al., 2014). Such findings suggest that resource depletion is not confined to a single context but rather exhibits generalizability and transferability. Several theoretical perspectives help explain this outcome.

First, the COR Theory posits that burnout reflects prolonged depletion of internal resources (Wendling et al., 2018). When individuals fail to replenish these resources through rest or effective emotional regulation, burnout is likely to occur. College athletes, facing dual pressures from training and academics, often lack sufficient time for recovery, making them more vulnerable to resource exhaustion. From a neurocognitive standpoint, chronic fatigue and sleep deprivation impair the functioning of the prefrontal cortex, which governs attention and emotional regulation (van der Linden et al., 2003). This impairment directly undermines individuals' ability to concentrate and manage academic demands. Furthermore, Recovery Theory emphasizes that in contexts of sustained pressure, a lack of effective recovery mechanisms keeps individuals' physiological and psychological systems in a prolonged state of arousal, thereby accelerating the depletion process (Balk and de Jonge, 2021).

In summary, the fatigue and resource depletion accumulated by college athletes during training may transfer into academic contexts, triggering persistent academic burnout. Therefore, future interventions aimed at alleviating academic burnout in college athletes should not only focus on enhancing support systems within academic settings but also emphasize the identification and management of cumulative load and fatigue risks within the athletic domain. Achieving coordinated recovery and resource integration across contexts is essential for promoting sustainable adaptation and wellbeing in this population.

### 4.2 The mediating role of emotion regulation ability

The results of this study indicate that emotion regulation ability (CR) plays a significant partial mediating role in the relationship between college athlete burnout and academic burnout, which is in line with H2. This suggests that college athlete burnout not only directly exacerbates academic burnout among individuals, but may also indirectly impair their academic adjustment by reducing their capacity for effective emotion regulation. This finding is consistent with previous related research results. For example, Liu et al. found that emotion regulation mediated the relationship between post-traumatic stress and negative emotions in breast cancer patients, indicating that individuals with poorer emotion regulation abilities were more likely to experience persistent negative emotional responses under high-stress conditions (Teng et al., 2022). Similarly, in a review of emotion regulation mechanisms in social anxiety and depression, Dryman and Heimberg noted that insufficient CR ability was strongly associated with higher levels of anxiety symptoms and depressive mood (Dryman and Heimberg, 2018). Despite differences in specific stressors and negative psychological outcomes, these studies consistently emphasize that emotion regulation ability serves as a core mechanism for individuals to adapt to stressful situations and plays a crucial mediating role in mitigating the adverse effects of stress.

The potential underlying mechanism can be explained by emotion regulation theory (Gross, 2015). According to this theory, CR is a proactive regulatory strategy that requires the engagement of executive functions in the prefrontal cortex to effectively reframe the meaning of stressful situations, thereby reducing negative emotional activation (Gross, 2015). However, the psychological and physiological fatigue associated with college athlete burnout may diminish the availability of cognitive resources in the prefrontal cortex, making it difficult for individuals to engage in effective cognitive restructuring. As a result, they struggle to regulate the negative emotions elicited by academic challenges (Kohn et al., 2014). Therefore, when college athletes are in a state of heightened fatigue, they are less able to employ adaptive emotion regulation strategies to cope with stress, and this decline in regulatory capacity directly weakens their positive psychological response to academic demands, ultimately leading to more severe experiences of academic burnout.

In summary, this study underscores the intervention value of enhancing emotional regulation abilities among college athletes. It is recommended that athletic programs and university psychological support systems incorporate emotion regulation training—such as CR exercises and mindfulness-based interventions—to strengthen individuals' cognitive coping skills and thereby alleviate the accumulation of burnout under the dual pressures of athletic training and academic demands.

### 4.3 The mediating role of sleep quality

This study found that sleep quality partially mediated the relationship between college athlete burnout and academic burnout, which is in line with H3, suggesting that athlete burnout not only directly undermines academic adjustment but may also indirectly exacerbate academic burnout by impairing physiological recovery mechanisms. This finding is highly consistent with existing research. Studies among Chinese college students have shown that sleep quality serves as a partial mediator between stress and academic burnout, indicating that sleep disturbances are a key pathway through which stress is transformed into burnout (Koutsimani et al., 2019). In medical student populations, research by Pagnin et al. revealed a significant bidirectional relationship between sleep disturbances and academic burnout, with poor sleep quality markedly increasing the risk of academic burnout (Pagnin et al., 2014). Furthermore, studies on healthcare professionals have demonstrated that sleep quality significantly moderates the pathway from occupational stress to physical and emotional health outcomes, supporting the view that sleep functions as a “restorative resource”—the depletion of which can lead to a cascade of psychological adaptation problems (Luo et al., 2025; Darydzaky and Desiana, 2023).

From a theoretical perspective, the mediating role of sleep quality in the transition from college athlete burnout to academic burnout can be explained through two primary pathways: physiological restoration and cognitive-neural resource regulation. First, sleep is a critical process for bodily recovery, with slow-wave sleep (SWS) playing an essential role in physical repair and psychological resource replenishment. Prolonged training loads and psychological stress can lead to sustained sympathetic nervous system activation, disrupting both the quality and duration of slow-wave sleep (Åkerstedt and Nilsson, 2003). Inadequate SWS not only impedes physiological recovery processes such as growth hormone secretion and glycogen replenishment but also interferes with the resetting of emotional regulation networks—particularly the prefrontal-amygdala circuitry—leading to diminished emotional control, increased affective instability, and cumulative negative mood states the following day (Walker and van Der Helm, 2009). Moreover, the cognitive impairments caused by insufficient sleep represent another critical pathway through which sleep quality mediates the relationship. Neuroimaging studies have demonstrated that sleep deprivation significantly reduces neural activity in the prefrontal cortex, particularly in the dorsolateral prefrontal cortex (dlPFC) and the anterior cingulate cortex (ACC), leading to widespread declines in attention, working memory, and executive functioning (Ma et al., 2015). This depletion of cognitive resources directly undermines college athletes' ability to sustain attention and maintain cognitive flexibility in academic settings, rendering them less capable of coping with ongoing academic demands, and ultimately intensifying their experience of academic burnout. Recent research has also shown that chronic poor sleep quality significantly predicts decreased academic self-efficacy and increased academic procrastination, both of which are hallmark manifestations of academic burnout.

In summary, sleep functions as a core restorative resource, linking athletic burnout to academic burnout through two critical pathways: physiological recovery and cognitive-emotional functioning. Accordingly, optimizing sleep quality among college athletes is not only essential for physical recovery in the athletic domain, but also represents a crucial intervention target for mitigating academic stress and reducing the risk of academic burnout.

### 4.4 The chain mediating role of emotion regulation and sleep quality

Finally, the present study confirmed the chain mediating role of emotion regulation (CR) and sleep quality in the relationship between college athlete burnout and academic burnout, which is in line with H4. This finding indicates that burnout among college athletes not only directly contributes to elevated levels of academic burnout, but also indirectly intensifies it by impairing emotion regulation capacity, which in turn disrupts sleep quality, thereby further exacerbating academic burnout. This result is consistent with previous research. For instance, Latif et al. found that higher levels of CR were significantly associated with better sleep quality in college student populations. Individuals who frequently employed CR strategies experienced fewer episodes of emotional rumination and anxiety before bedtime, thereby improving their overall sleep quality (Latif et al., 2019). Moreover, the review by Vandekerckhove and Wang further demonstrated the complex and bidirectional interactions between emotion regulation and sleep quality. Specifically, poor cognitive emotion regulation not only contributes to difficulties falling asleep but also reduces the proportion of slow-wave sleep (SWS) in the sleep cycle, ultimately impairing physiological recovery and cognitive functioning (Vandekerckhove and Wang, 2017).

This chain mediation pathway may reflect a progressive depletion of psychological resources, wherein impaired emotion regulation serves as the initial trigger, leading to disrupted sleep functions and ultimately exacerbating academic maladjustment. First, under the condition of college athlete burnout, individuals frequently encounter frustration, emotional instability, and diminished self-control. This state impairs their capacity to implement effective CR strategies, thereby leading to greater emotional burden in daily life (Gross, 2015). Persistent emotional exhaustion has been identified as a key antecedent of sleep disturbances, including difficulties with sleep onset, reduced proportions of slow-wave sleep, and increased nocturnal awakenings (Palmer and Alfano, 2017). Second, emotional dysregulation-induced hyperarousal persistently activates the hypothalamic–pituitary–adrenal (HPA) axis and the sympathetic nervous system, leading to elevated cortisol levels, which hinder both the initiation and maintenance of sleep (Baglioni et al., 2010). Once sleep quality deteriorates, individuals tend to exhibit impaired attention, executive functioning, and emotional resilience the next day (Krause et al., 2017), further weakening their capacity to cope with academic stress. This, in turn, exacerbates psychological exhaustion and cognitive withdrawal. This mechanism is particularly pronounced under chronic stress conditions, forming a progressive chain of “emotional dysregulation → sleep disturbance → functional exhaustion.”

In summary, this chain mediation pathway suggests that under the dual pressures of athletic training and academic demands, emotional dysregulation not only exerts a direct impact but also amplifies its effect on academic burnout through the restorative system of sleep. This underscores the dynamic coupling between regulatory capacity and recovery mechanisms. Notably, although the chain mediation effect reached statistical significance, its effect size was relatively small, indicating that its practical impact in intervention settings may be limited. Therefore, it is advisable to consider this pathway in conjunction with other more practically significant mechanisms when designing interventions.

### 4.5 Limitations and future research

Despite providing new empirical evidence for the mediating pathways through which college athlete burnout influences academic burnout via emotional regulation and sleep quality, this study has several limitations that should be addressed in future research. First, the cross-sectional design limits the ability to infer causal relationships and capture the temporal dynamics among variables. Emotional regulation ability, sleep quality, and burnout levels may fluctuate with changes in training schedules and academic periods. Therefore, future studies could adopt longitudinal tracking or experience sampling methods (ESM) to more precisely depict the evolving mediation patterns over time. Second, the study primarily relied on self-reported questionnaires, which may be subject to social desirability bias and common method variance. This concern is particularly salient among athlete populations, where individuals might underreport fatigue or emotional issues to maintain a favorable self-image. It is recommended that future research incorporate teacher or coach evaluations, as well as physiological indicators (e.g., heart rate variability, sleep monitoring) to enhance measurement objectivity and ecological validity. Lastly, the study focused solely on cognitive reappraisal as a single emotion regulation strategy, without considering the multidimensional nature or flexibility of emotion regulation. This may limit a comprehensive understanding of the underlying mechanisms.

In addition to the limitations noted above, the findings of this study provide several practical implications. First, the significant mediating roles of emotion regulation and sleep quality highlight the importance of integrating psychological skills training and sleep management programs into athletic and academic support services. Universities and sports organizations should consider developing targeted interventions, such as cognitive reappraisal training and sleep hygiene education, to help college athletes cope with dual stressors more effectively. Second, the identification of a chain mediation pathway suggests that holistic interventions addressing both emotional regulation and restorative recovery mechanisms may be particularly beneficial for reducing academic burnout in athletes.

Future research could build upon these findings in several ways. Longitudinal designs are needed to establish the causal directionality of these relationships, while experimental studies could test the effectiveness of specific interventions (e.g., mindfulness-based training or structured sleep programs). Moreover, cross-cultural studies are recommended to examine whether these mechanisms generalize across different educational and athletic systems.

## 5 Conclusion

This study investigated the relationship between college athlete burnout and academic burnout, with a focus on the multiple mediation pathways involving emotional regulation ability (cognitive reappraisal) and sleep quality. The findings offer both theoretical contributions and practical implications, and the main conclusions are as follows:

(1) College athlete burnout significantly and positively predicts academic burnout.
(2) Emotional regulation ability (cognitive reappraisal) plays a partial mediating role in the relationship between college athlete burnout and academic burnout.
(3) Sleep quality also serves as a mediator between college athlete burnout and academic burnout.
(4) Emotional regulation ability (cognitive reappraisal) and sleep quality jointly form a significant chain mediation pathway linking college athlete burnout and academic burnout.

Overall, this study deepens our understanding of the connection between college athlete burnout and academic burnout, and provides a scientific foundation for academic management and psychological health interventions targeting college athletes.

## Data Availability

The original contributions presented in the study are included in the article/Supplementary material, further inquiries can be directed to the corresponding authors.
